# Longitudinally Extensive Transverse Myelitis: A Retrospective Study Differentiating Neuromyelitis Optica Spectrum Disorder From Other Etiologies

**DOI:** 10.7759/cureus.13968

**Published:** 2021-03-18

**Authors:** Sunanda Paudel, Gaurav Nepal, Sandesh Guragain, Sangam Shah, Basanta S Paudel, Rajeev Ojha, Reema Rajbhandari, Ragesh Karn, Bikram P Gajurel, Sunanda Paudel

**Affiliations:** 1 Neurology, Tribhuvan University Teaching Hospital, Kathmandu, NPL; 2 Internal Medicine, Tribhuvan University Institute of Medicine, Kathmandu, NPL; 3 Neurology, Nepal Medical College Teaching Hospital, Kathmandu, NPL; 4 Neurology, Maharajgunj Medical Campus, Kathmandu, NPL; 5 Neurology, Tribhuvan University Institute of Medicine, Kathmandu, NPL; 6 Vascular Neurology, Tribhuvan University Teaching Hospital, Kathmandu, NPL; 7 Neurology, Institute of Medicine, Tribhuvan University Teaching Hospital, Kathmandu, NPL

**Keywords:** longitudinally extensive transverse myelitis, letm, nmosd, aquaporin-4, neuromyelitis optica spectrum disorder

## Abstract

Background

Longitudinally extensive transverse myelitis (LETM) is characterized by contiguous immune-mediated inflammatory lesion of the spinal cord extending more than three vertebral segments. Neuromyelitis optica spectrum disorder (NMOSD) is the most common and important cause of LETM. This study aims to evaluate the demographic profile, clinical presentations, neuroimaging features, laboratory parameters, and etiologies of LETM and differentiates NMOSD from other etiologies of LETM.

Methodology

This retrospective cross-sectional study was conducted at the Department of Neurology, Tribhuvan University Teaching Hospital, Kathmandu, Nepal. After receiving clearance from the ethical committee, a retrospective chart review was conducted and records of all the inpatient LETM cases admitted from March 2018 to June 2020 were obtained. From the patient records, the following information was extracted: the demographic profile, clinical presentations, neuroimaging features, cerebrospinal fluid analysis, serum anti-aquaporin-4 (AQP4) immunoglobulin G status, hemogram, infectious disease profile, inflammatory markers, and auto-immune panels. Descriptive analysis of data was performed with SPSS statistics 23.0 version (IBM Corp, Armonk, NY, USA).

Results

In our study, the mean age of LETM patients was 36.58 years, and 12 out of 19 (63.15%) patients were young, with an age less than 40 years. A total of 13 (68.40%) patients were male, with a male-to-female ratio of 2.16. Seven (36.80%) patients had a clinical diagnosis of NMOSD with anti-AQP4 antibody-positive status, four (21.10%) had unknown etiology, three (15.8%) had post-infectious transverse myelitis, and three (15.80%) had a diagnosis of idiopathic transverse myelitis. There was a single case (5.30%) of cervical spondylotic myelopathy and leukemic transverse myelitis each. The common presenting symptoms of LETM were bladder dysfunction, paraparesis, quadriparesis, and visual impairment. Visual involvement, either unilateral or bilateral, was common in NMOSD and LETM of unknown etiology category. Similarly, brain lesion was common in patients with NMOSD and LETM of unknown etiology category.

Conclusion

LETM is a heterogeneous disorder with diverse etiologies and clinical presentations. NMOSD is an important cause of LETM that predominantly affects females. Optic neuritis can be seen in LETM of various etiologies, but it is more common in anti-AQP4-positive NMOSD patients. Brain lesions in LETM are common in anti-AQP4-positive NMOSD.

## Introduction

Longitudinally extensive transverse myelitis (LETM) is an entity with a substantial spinal cord lesion that spans three or more vertebral segments on spinal magnetic resonance imaging (MRI) [1]. Neuromyelitis optica spectrum disorder (NMOSD) invariably is the most common cause of LETM [2]. However, multiple sclerosis (MS), myelin oligodendrocyte glycoprotein antibody disorders (MOGAD), acute disseminated encephalomyelitis (ADEM), glial fibrillary acidic protein (GFAP) astrocytopathy, spinal cord infarction, parainfectious myelopathy, neurosarcoidosis, Sjögren syndrome, systemic lupus erythematosus, neuro-Behçet disease, paraneoplastic myelitis, and dural arteriovenous fistula are also known to cause LETM [2].

The NMOSD characteristically involves the optic nerve, spinal cord, area postrema, brainstem, diencephalon, or cerebrum, which bestow the core clinical characteristics of NMOSD. Nevertheless, the aforementioned conditions can easily mimic NMOSD as they might also involve optic nerves and/or spinal cord, presenting with bilateral optic neuritis and LETM. The above-mentioned conditions may even have brain lesions resembling those of NMOSD [3]. Therefore, it is imperative to exclude all causes of LETM before diagnosing NMOSD. As NMOSD is one of the disabling diseases of the central nervous system, early diagnosis and management are essential for optimal outcome and better quality of life [3,4].

As mentioned above, LETM has an important and characteristic association with NMOSD, and almost half of the adult LETM cases are due to NMOSD [5]. NMOSD is found in all races around the world, and recent population-based studies have shown that NMOSD is more common in non-white races. Studies published in Asia show that the prevalence of NMOSD has increased recently, and the ratio of NMOSD to MS is higher compared with Western countries [6,7]. Nevertheless, in the medical literature, there are no clinical studies on LETM from Nepal, an Asian country. Therefore, we conducted a retrospective study in our tertiary care center to evaluate the demographic characteristics, clinical manifestations, neuroimaging characteristics, laboratory parameters, and etiology of LETM patients.

## Materials and methods

This study was approved by the Institutional Review Board (IRB) of Tribhuvan University Institute of Medicine (approval number, 34/ (6-11) E2/ 077/ 078). We conducted this retrospective cross-sectional study at the Department of Neurology, Tribhuvan University Teaching Hospital (TUTH). TUTH, located in the capital city of Kathmandu with 700 beds and 32 departments, is the largest hospital in the country and a tertiary referral center for all kinds of diseases and conditions including neurological disorders. After receiving clearance from the ethical committee of the IRB, a retrospective chart review was done. Records of all inpatient LETM cases admitted from March 2018 to June 2020 were obtained. The inclusion criteria were extensive longitudinal involvement of three or more segments of the spinal cord on MRI in patients aged ≥16 years.

From the patient records, the following information was extracted: demographic profile, clinical presentations, neuroimaging features (MRI brain and spine with T1-weighted, T2-weighted, fluid-attenuated inversion recovery, diffusion-weighted imaging, and gadolinium contrast images), cerebrospinal fluid (CSF) analysis for cytology, biochemistry and oligoclonal bands, and serum anti-aquaporin-4 (AQP4)-immunoglobulin G (IgG) status. Other non-specific investigations done were complete blood count, serum vitamin B12 level, thyroid function tests, infectious disease profile, inflammatory markers (erythrocyte sedimentation rate and C-reactive protein), and auto-immune panel including antinuclear antibodies, anti-double-stranded DNA antibodies, anti-nucleosome, anti-histones, anti-Sm, anti SS-A, anti-SS-B, anti-RO, anti-Scl-70, anti RibP-protein, and anti-JO. Patient records with incomplete routine evaluation, neuroimaging features, CSF reports, infectious, inflammatory, and auto-immune profiles were excluded. NMOSD was diagnosed using the 2015 American Academy of Neurology international consensus diagnostic criteria [8]. The 2017 revisions of the McDonald criteria were used to diagnose MS [9].

With proper evaluation of clinical, neuroimaging, and laboratory findings, patients were divided into three diagnostic categories: NMOSD, LETM of unknown etiology, and other causes of LETM. Descriptive analysis was performed and various study variables were compared among these three groups. Descriptive analysis of data was performed using SPSS statistics 23.0 version (IBM Corp, Armonk, NY, USA).

## Results

A total of 19 patients were included in this study. The mean age of the patients was 36.58 years with a standard deviation of 19.33. Twelve out of 19 patients (63.15%) were young, with an age of less than 40 years. Of the total number of patients, 13 (68.40%) were male, with a male-to-female ratio of 2.16. A total of four (21.10%) patients had a history of antecedent respiratory infection. Nine (47.40%) patients had symptoms that progressed over hours, and the remaining 10 (52.60%) patients had progression over days (Table 1).

**Table 1:** The demographic profile of patients with LETM. LETM, longitudinally extensive transverse myelitis

Variables	Frequency (%)
Age group (Years)	
16-20	5 (26.3%)
21-40	7 (36.8%)
41-60	4 (21.1%)
61-80	3 (15.8%)
Sex	
Male	13 (68.4%)
Female	6 (31.6%)
Antecedent infection	
Present	4 (21.1%)
Absent	15 (78.9%)
Course of symptoms	
Progressed in hours	9 (47.4%)
Progressed in days	10 (52.6%)

Out of the total of 19 LETM patients, seven (36.80%) had a clinical diagnosis of NMOSD with anti-AQP4 antibody-positive status, four (21.10%) had unknown etiology, three (15.8%) had post-infectious transverse myelitis, and three (15.80%) had a diagnosis of idiopathic transverse myelitis. There was a single case (5.30%) of cervical spondylotic myelopathy and leukemic transverse myelitis each (Table 2).

**Table 2:** Diagnostic category of patients with LETM. LETM, longitudinally extensive transverse myelitis; NMOSD, neuromyelitis optica spectrum disorder

Diagnosis	Number (%) of patients
NMOSD	7 (36.84%)
Post-infectious transverse myelitis	3 (15.78%)
Idiopathic transverse myelitis	3 (15.78%)
Leukemic transverse myelitis	1 (5.26%)
Unknown etiology	4 (21.05%)
Cervical spondylotic myelopathy	1 (5.26%)

A total of five (45.45%) patients with NMOSD had paraparesis and two (25%) had quadriparesis. Of the 19 patients with LTEM, 15 (79%) had some form of bladder involvement. A total of 11 (57.89%) patients had visual impairment. Six patients had bilateral visual impairment and four (66.70%) were proven to have NMOSD. Of the seven patients with NMOSD, six (85.71%) had CSF pleocytosis. All the patients with LETM of unknown etiology had CSF pleocytosis, whereas two (28.57%) out of the seven patients with other etiology of LETM had CSF pleocytosis. All patients with NMOSD and unknown LETM etiology had elevated CSF protein levels, whereas seven (87.50%) patients with other LETM etiology had elevated CSF protein levels. Brain lesions were seen in three of six (50%) patients with NMOSD. Two (33.30%) patients with unknown etiology and only one patient with other LETM etiology had brain lesions. MRI revealed cervical cord involvement in six (31.57%) patients, 10 (52.63%) patients had thoracic cord involvement, and three (15.78%) patients had both cervical as well as thoracic cord involvement. A total of two (33.33%) patients with NMOSD had cervical cord involvement whereas one (16.67%) patient with unknown LETM etiology and three (50%) patients with other LETM etiology had cervical cord involvement. Four (40%) patients with NMOSD, two (20%) with unknown etiology, and four (40%) with other LETM etiology had thoracic cord involvement. Only one (33.33%) patient in each diagnostic category had both cervical and thoracic cord involvement (Table 3).

**Table 3:** Comparison of clinical, laboratory, and imaging features of NMOSD with other causes of LETM. AQP4, aquaporin-4; CSF, cerebrospinal fluid; LETM, longitudinally extensive transverse myelitis; NMOSD, neuromyelitis optica spectrum disorder

Variables	AQP4-positive NMOSD (n = 7)	Unknown etiology of LETM (n = 4)	Other LETM (n = 8)
Age group			
0-20	1 (20%)	0 (0%)	4 (80%)
21-40	2 (28.57%)	3 (42.86%)	2 (28.57%)
41-60	2 (50%)	1 (25%)	1 (25%)
61-80	2 (66.67%)	0 (0%)	1 (33.33%)
Sex			
Male	2 (15.38%)	4 (30.76%)	7 (53.84%)
Female	5 (83.33%)	0 (0%)	1 (16.67%)
Weakness pattern			
Quadriparesis	2 (25%)	3 (37.50%)	3 (37.50%)
Paraparesis	5 (45.45%)	1 (9.10%)	5 (45.45 %)
Bladder involvement	5 (33.33%)	3 (20%)	7 (46.67%)
Visual involvement			
Unilateral	2 (40%)	2 (40%)	1 (20%)
Bilateral	4 (66.7%)	1 (16.67%)	1 (16.67%)
CSF cell count (/mm^3^)			
<5	1 (16.67%)	0 (0%)	5 (83.33%)
6-50	4 (50%)	2 (25%)	2 (25%)
51-100	2 (50%)	2 (50%)	0 (0%)
Cerebrospinal fluid protein (mg%)			
46-100	1 (20%)	0 (0%)	4 (80%)
>100	5 (41.67%)	4 (33.33%)	3 (25%)
Brain lesion	3 (50%)	2 (33.33%)	1 (16.67%)
Spinal cord lesion			
Cervical	2 (33.33%)	1 (16.67%)	3 (50%)
Thoracic	4 (40%)	2 (20%)	4 (40%)
Cervical and thoracic	1 (33.33%)	1 (33.33%)	1 (33.33%)

## Discussion

In our study, of the total 19 patients, seven (36.84%) had AQP4 antibody-positive NMOSD, while the remaining patients had other diagnoses that included unknown etiology, post-infectious transverse myelitis, idiopathic transverse myelitis, leukemic transverse myelitis, and cervical spondylotic myelopathy.

However, no LETM patients in our study had a diagnosis of MS. Although MS is one of the most common neurological diseases of the central nervous system, it is unevenly distributed worldwide. According to the 2016 Global Burden of Disease Study, in North America and Europe, the age-standardized MS prevalence rate is higher than 120 cases per 100,000 population; in Australasia, it is moderate (60-120 per 100,000 population); and the lowest (<60 per 100,000 population) prevalence is seen in North Africa, Middle East, Latin America, Asia, Oceania, Caribbean, and Sub-Saharan Africa. The low prevalence of MS in Asia may explain our findings, which showed no cases of MS among LETM patients [10]. Likewise, we encountered no cases of ADEM in our study. ADEM is predominantly a childhood disorder and rare in older adults. Because we included LETM patients with ages greater than or equal to 16, all cases of ADEM were likely excluded [11].

Similarly, there were no cases of MOGAD in our study sample. MOGAD are immune-mediated inflammatory disorders of the central nervous system that can have similar manifestations as NMOSD. The prevalence of MOGAD has not been studied in-depth, and the exact prevalence is unclear. Previously, MOGAD and NMOSD were considered similar disease entities. Indeed, a small number of AQP4-IgG seronegative NMOSD patients tested positive for MOG-Ab. However, because the spectrum of MOGAD covers many atypical NMOSD manifestations, and due to the pathophysiological differences (NMOSD is astrocytopathy, while MOGAD is oligodendrocytopathy), there is an increasing tendency to recognize NMOSD and MOGAD as distinct entities [4,12].

In our study, all patients diagnosed with NMOSD were seropositive for AQP4-IgG. AQP4 water channels are widely expressed in astrocytes of the brain, spinal cord, and optic nerves. Based on cell-based assays, the sensitivity of serum AQP4-IgG to NMOSD is 76.7% and the specificity is 99.8% [13]. Although most patients with NMOSD have serum anti-AQP4-IgG, NMOSD can still be diagnosed with seronegative or unknown status of AQP4-IgG [8].

In our study, four (21.05%) patients had a diagnosis of LETM of unknown etiology. In these patients, no definite diagnosis could be made through existing investigations. Non-availability of some molecular investigations, unaffordability of investigations by poor patients, and lack of patient follow-up were the reasons we could not determine the possible etiologies in these LETM patients. We believe these etiologies could be either post-infectious, autoimmune, or paraneoplastic forms of demyelinating diseases [14].

In our study, common presenting symptoms were bladder dysfunction, paraparesis, quadriparesis, and visual impairment. Because the underlying etiology of LETM is difficult to determine, each case should be properly evaluated both clinically and with elaborate laboratory investigations to look for any subtle clues. Visual involvement, either unilateral or bilateral, was common in NMOSD and LETM of unknown etiology category. Although optic neuritis attacks in NMOSD are mostly unilateral, sequential optic neuritis or simultaneous optic neuritis of both eyes favor the diagnosis of NMOSD [15]. Brain lesion was seen in three (42.86%) of the seven patients with NMOSD. Whereas two (50%) out of the four patients with unknown etiology and only one (12.5%) patient with other etiologies had brain lesions. With the discovery of AQP4-IgG testing, it is evident that a significant proportion of patients with NMOSD had brain MRI abnormalities, seen in the areas where the expression of AQP4 was high. However, it is not rare to have abnormalities in other sites where the expression of AQP4 is not high [16].

In our study, six (85.71%) out of the seven patients with NMOSD had CSF pleocytosis, and all seven with NMOSD had raised protein levels. CSF abnormalities are common during the acute attack of NMOSD and can have pleocytosis and elevated protein levels [8]. Although CSF pleocytosis or enhancement of lesions on MRI has been widely used to define inflammatory myelopathy, similar features have been reported in non-inflammatory myelopathy. In a wide range of myelopathy, the overlap of clinical, MRI, and CSF features may lead to a false diagnosis of LETM. A study conducted in patients with transverse myelitis showed that CSF pleocytosis is common in inflammatory myelopathy (NMOSD, MS, MOGAD, and ADEM). However, CSF pleocytosis is also present in a considerable number of vascular myelopathy patients. The same study showed that elevated CSF proteins are more frequently observed in vascular myelopathy and spondylotic myelopathy. CSF oligoclonal bands were almost exclusive of inflammatory myelopathy [17].

Although this is the first study of its kind to explore the etiology of LETM in Nepal, our study has many limitations worth mentioning. The most important limitation of this study is the retrospective design and small sample size. Furthermore, financial constraints restricted various laboratory and imaging tests for LETM patients, which were later classified as LETM of unknown etiology.

## Conclusions

LETM is a heterogeneous disorder with diverse etiologies and clinical presentations. NMOSD was the most common cause of LETM followed by post-infectious and idiopathic transverse myelitis. Bilateral optic neuritis is more common in anti-AQP4-IgG-positive NMOSD compared to other etiologies of LETM. Brain lesions at different locations can be seen in LETM regardless of etiology, but they more common in anti-AQP4-IgG-positive NMOSD.
